# Effect of zinc substitution in hydroxyapatite coating on osteoblast and osteoclast differentiation under osteoblast/osteoclast co-culture

**DOI:** 10.1093/rb/rbz001

**Published:** 2019-02-08

**Authors:** Guolong Meng, Xiaoli Wu, Ruijuan Yao, Jing He, Wu Yao, Fang Wu

**Affiliations:** National Engineering Research Center for Biomaterials, Sichuan University, Chengdu 610064, P.R. China

**Keywords:** zinc, bone marrow stromal cell, osteoclast precursor, osteoclast, co-cultivation, bone remodelling process

## Abstract

Zinc is an essential trace element required for bone remodelling process, but its role in such process remains to be elucidated. In particular, inconsistent results have been reported on the effect of Zn on osteoclastic responses, and supplement of receptor activator of nuclear factor kappa-B ligand (RANKL) factors has been commonly adopted. Co-culture is a suitable approach to elucidating the role of Zn in bone remodelling process, by better imitating the cellular environment as the presence of osteoblasts plays critical role in modulating osteoclastic functions. In this study, zinc-substituted HA coatings have been deposited using a liquid precursor plasma spraying process at two different concentrations (1, 2 wt.%). The effect of zinc substitution on osteoblastic and osteoclastic differentiation has been studied *in vitro*. In particular, a cultivation regime was designed to first induce osteoblastic differentiation of rat bone marrow stromal cells (BMSCs) for 14 days, and then induce osteoclastic differentiation of osteoclast-like precursor RAW 264.7 cells through the aid of the osteoblasts formed for additional 14 days, in the absence of the external addition of RANKL. The results showed that Zn substitution moderately promoted the BMSC differentiation into the osteoblasts and reduced the osteoclastic activity in early time (1 day co-culture). However, promotion of the osteoclastic activity were observed at later stages, as indicated by the significantly enhanced expressions of trap5b and IL-1 (8- and 15-day co-culture) and moderate stimulation of the nucleus integration and formation of the multinucleated cells (14-day co-culture). Such stimulating effect of the osteoclastic activity was absent under mono-culture of RAW 264.7 cell, with simple RANKL supplementation. The results suggest that both the zinc and the presence of MSC/osteoblast play profound and highly interacted roles on osteoclast differentiation and activity, which is critical in modulating the bone remodelling process.

## Introduction

Bone mass is maintained by repeating cycle of destruction and rebuilding to keep the balance between bone resorption and formation [1]. The regulation of hormones and other molecules secreted by osteoblast (OB) and osteoclast (OC) control the dynamic and complex process of bone formation and reconstruction [2, 3].

Zn is an essential trace element to metabolism function. Zn deficiency reduces bone weight, delays bone metabolism and results in retardation of bone growth [4–6]. Zinc has a stimulatory effect on bone formation and mineralization *in vitro* and *in vivo* [7, 8]. Zn stimulated cell proliferation and differentiation, as well as protein synthesis in osteoblastic MC3T3-E1 cells [9–11]. The cellular mechanism of zinc function in OCs has not been fully understood. Zn has been generally reported to inhibit osteoclastic differentiation [12, 13]. For instance, Yamada *et al*. [12] showed a composition dependent increase in OC apoptosis, where the number of apoptotic OCs was 6.3 times higher with Zn-incorporated (0.633 wt. %) tricalcium phosphate ceramic (TCP) than that with the pristine TCP after 1-day culture. However, inconsistent results have been reported in the literature [14–17]. Holloway *et al*. [16] reported that Zn increases the tartrate-resistant acid phosphatase (TRAP) positive cell number and simultaneously inhibit resorption after 1 day in co-culture of OBs and OCs.

Therefore, it is important to clarify the role of Zn in osteoblastic and osteoclastic differentiation. Previous studies have often used receptor activator of nuclear factor kappa-B ligand (RANKL) supplementation to study the effect of Zn on OC differentiation and activity [13]. The OB itself, however, plays a critical role in modifying the OC differentiation and function in the natural process. The dominating pathway regulating the OC differentiation is the RANKL/RANK/osteoprotegerin (OPG) pathway, where the presence of the OB promotes OC differentiation through membrane presentation of RANKL and its binding to the membrane receptor RANK on mononuclear OC precursors [3].

Recently, co-culture approach has been increased used for the biological studies of biomaterials, since the cellular environment in co-culture condition is closer to *in vivo* compared with mono-culture, with appropriate cell–cell interactions. Heinemann *et al*. [18] reported a human co-culture model including both OCs and OBs to imitate *in vivo* condition. It has been reported that the osteocytes and OBs act synergistically to stimulate the osteogenic differentiation of MSCs under co-culture condition [19].

The objective of present study was to understand the role of Zn substitution in HA on OB and OC responses under mono-culture and co-culture conditions, and in particular the differentiation and activity of OC in the presence and the absence of the OB. To this aim, BMSC induced OB were co-cultivated with OC-like-cell precursor cell RAW 264.7 to induce it into OC, without the external addition of RANKL. To examine if and how cell–cell interactions would have a synergetic role with Zn element in affecting the cell behaviours, we used both co-culture and mono-culture conditions to monitor the effect of cellular environment on the modulating role of ZnHA on the proliferation and differentiation of OB and the differentiation of OC. This study provides important insights into the mechanisms of Zn regulating the OB and OC differentiation.

## Materials and methods

### Materials

The HA liquid precursors were prepared by the wet chemical synthesis, through a reaction between Ca(NO_3_)_2_·4H_2_O (Kelong Corp., Chengdu) and (NH_4_)_2_HPO_4_ (Kelong Corp.) in PH = 10 alkaline solution. The starting Ca/P molar ratio was equal to the stoichiometric value of HA. In brief, the liquid precursors were prepared by gradually dropped the (NH_4_)_2_HPO_4_ aqueous solution into the Ca(NO_3_)_2_ solution. All reactions were stirred and kept at 70°C and adjusted the pH value to 10 by the addition of the NH_3_·H_2_O slowly. The liquid precursors were aged at 70°C for 24 h. The Zn-HA (1% and 2% wt. Zn) precursors were prepared used the same procedure described above, except that desired amounts of Zn(NO_3_)_2_ solutions (Kelong Corp.) were added into the Ca(NO_3_)_2_ solution first, with a (Ca + Zn)/P atomic ratio of 1.67.

The HA and 1%, 2% Zn-HA coatings were deposited on Ti–6Al–4V alloy substrates (Ф14 × 2mm) by the liquid precursor plasma spraying (LPPS) process, using the liquid precursors as feedstocks for plasma spraying. The LPPS was carried out using Metco MN air plasma spraying system (Metco Ltd., USA). The liquid precursors were transported by a peristaltic pump and were injected into the plasma jet through an atomizing nozzle. More detailed information of the LPPS process can be found elsewhere [20, 21].

### Coating characterization

Phase compositions of the HA and ZnHA coatings were characterized by XRD (DX-1000 CSC, China) with Cu Kα radiation, at operating conditions of 40 kV and 25 mA. The 2θ range is from 10° to 60°. Phases were identified by comparing the diffraction patterns with International Centre for Diffraction Data (ICDD) standards (PDF cards) using Jade 5.0 software.

The concentrations of Zn in cell suspension were performed using a flame atomic absorption spectrometer (Model GGX-6, Beijing Haiguang Instrument Co., Beijing, China) with Zeeman background correction.

### Cell co-culture and mono-culture on HA and ZnHA coatings

**Figure 1:**
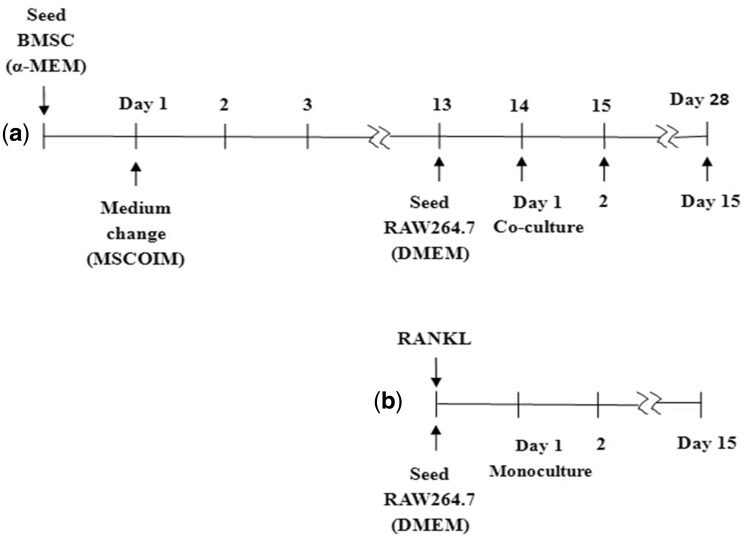
Schema of co-culture of BMSC/OB and RAW264.7/OC (**a**) and mono-culture of RAW264.7 with RANKL supplementation (**b**)

The HA and ZnHA coatings were gamma-sterilized at 25 kGy for cell experiments. The BALB/c BMSC (Batch number (MUCMX-90011), Cyagen corp., Guangdong, China) cell suspensions (2 × 10^4^ cells/ml) were seeded into 24-well plates. The BMSCs come from the bone marrow of BALB/c mouse. Induction of the osteogenic differentiation was initiated right after the first day by addition of the 100 nM dexamethasone (D1756, Sigma), 10 mM β-glycerophosphate (G9422, Sigma) and 50 μM Vitamin D3 (47763, Sigma) to the α-MEM medium supplemented with 10% fetal bovine serum (HyClone), 100 U/ml penicillin and 100 μg/ml streptomycin in a humidified atmosphere at 37°C and 5% CO_2_. The osteogenic induction medium for BMSC was marked as MSCOIM in Fig. 1. On Day 13, the OC-like-cell precursor Raw264.7 (TCM13, Chinese Academy of Science Cell Resource Center, Shanghai, China) cell suspension with 1× 10^5^ cells/ml was seeded on the top of the samples. The RAW264.7 comes from the ascites of BALB/c mouse. Co-culture was initiated on Day 13 and medium was changed to DMEM supplemented with 10% foetal bovine serum and 100 U/ml penicillin and 100 μg/ml streptomycin. The medium was renewed every 2 days. The schema for the co-culture and mono-culture are shown in Fig. 1, respectively. The two timescales used in Fig. 1a represents the total cultivation time for BMSC/OB and the cultivation time for RAW264.7/OC (in co-culture), respectively. From Day 14/1 up to Day 28/15, both OB and OC were cultivated on the coating samples. This resulted in a final 28 day culture for BMSC/OB and 15 day culture for the RAW264.7/OC, with the last 14 days for co-culture. For comparison, mono-culture of RAW264.7 with the supplement of 20 ng/ml (600 pmol/l) RANKL was carried out on samples under similar conditions (Fig. 1b).

### Immunofluorescence and confocal laser-microscopy

After the OC-like-cells Raw264.7/OC being mono-cultured for 12 days, the cells were dual-labelled with phalloidin for F-actin and for the vitronectin with monoclonal antibody vitronectin. Firstly, for labelling with vitronectin, the samples were washed with PBS and fixed in 3.7% paraformaldehyde solution, pH = 7.4 and at 25°C for 10 min. The samples were permeabilized using 1% Triton X-100/PBS for 15 min and unspecific binding was blocked with 1% BSA/PBS. Cells were incubated with the primary antibody (1:50 dilution in 0.1% BSA/PBS; Monoclonal anti-vitronectin antibody produced in rabbit; Bioss, China) for 30 min at 25°C. The secondary antibody (FITC conjugated goat anti-rabbit IgG) was diluted in TBST at a 1:100 dilution and was used to incubate the cells for 1 h. Secondly, the samples were also stained with phalloidin Alexa 594 (diluted in PBS 1:100, Sigma) for 30 min in the dark. Finally, for observing the nucleus, the cells were stained in 1 μg/ml Hoeschst33258/PBS (Sigma) solution for 5 min. After two additional washes with PBS, the samples were visualized using a confocal microscope (Leica. SP5, Germany). The cells in co-culture (14 days) were stained using the same above steps. The cell morphologies under mono-culture and co-culture conditions were also observed using the SEM (S4800, Tecnai F20, Tokyo, Japan).

### Enzyme-linked immunosorbent assay

OB and OC differentiation were evaluated by the measurement of ALP/COL I ratio and TRAP 5b activity, respectively. Cell proliferation was determined through the total activity of lactate dehydrogenase (LDH) in the cell lysates and methyl thiazolyl tetrazolium (MTT) assay. After incubation for 2, 4, 7, 14, 21 and 28 days, 200 μl MTT solution (5 μg/μl in PBS) was added to each well and incubated for another 4 h. The alkaline phosphatase (ALP), osteocalcin (OCN), collagen type I (Col I), LDH, interleukin IL-1, tumour necrosis factor alpha (TNF-α), parathyroid hormone (PTH), RANK ligand (RANKL), TRAP 5 b activity were measured at the prescribed culture times, using the quantitative enzyme-linked immunosorbent assay (ELISA). All the experiments were conducted strictly following the instructions of the ELISA kits (BYE30029 (ALP), BYE98007 (OCN), BYE30235 (COLI), BYE30583 (IL-1), BYE40025 (TNF-α), BYE30362 (PTH), BYE30442 (RANKL), BangYi, Shanghai, China).The expressions were determined by the absorbance measurements performed with a spectrophotometer (MK3, Thermo Electron Ltd, USA) at 450 nm, by comparing the measured OD values to the standard curve plotted using a set of standard samples.

### Statistical analysis

All measurements were collected at triplicate and expressed as means ±standard deviations. All results were statistically evaluated by ANOVA and SPSS statistical software. Analysis of variance was employed to assess significance.

## Results

### X-ray diffraction

**Figure 2:**
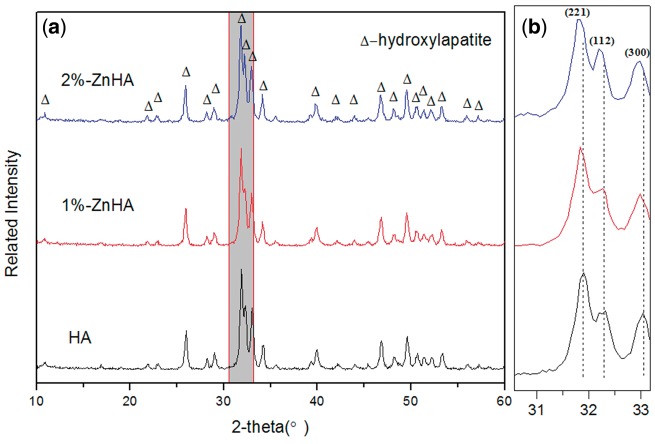
X-ray spectra of the pristine HA and two ZnHA samples. (**a**) The full spectra; (**b**) The blowup of the left spectra showing the shifting of the (2 2 1), (1 1 2) and (3 0 0) diffraction peaks, indicating the incorporation of Zn into the HA lattice and the formation of the solid solution

Zinc can be incorporated into hydroxyapatite crystal structures by replacing calcium ions. Figure 2 shows the XRD spectra of the pristine HA and two zinc-substituted HA (1% and 2%-Zn) coatings. The two ZnHA coatings showed well-resolved diffraction patterns consistent with the pristine HA (matching the ICDD standard PDF No. 74-0566). No peak indicating the presence of the secondary phases was observed for all three HA coatings. However, shifting of the (2 2 1), (1 1 2) and (3 0 0) diffraction peaks were clearly observed for the zinc-substituted HA coatings (with a further shifting to the left for 2%-ZnHA compared with the 1%-ZnHA) especially in the blowup spectra (Fig. 2b), indicating the successful incorporation of Zn in the HA lattice and the formation of the solid solution. X-ray spectra of the ZnHA coatings before and after soaking in cell culture medium (7, 14, 28 days) were also examined. The results (Fig. 4D) suggest both ZnHA coatings are quite stable, with no indication of phase transformations observed.

### Cell behaviours of OBs

A cultivation regime was adopted to firstly induce the osteoblastic differentiation of the mouse bone marrow stromal cells using the osteogenic induction medium, followed by the induction of osteoclastic differentiation of OC precursors RAW264.7 through the aid of the BMSC transformed OBs, without the addition of the external factors of RANKL (Fig. 1a).

**Figure 3:**
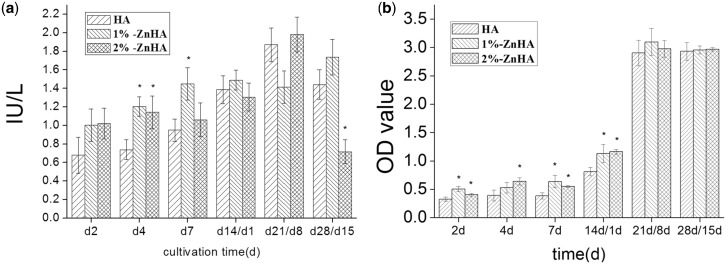
(**a**) LDH activity of the BMSC/OC measured under mono-culture (0‒13 days) and co-culture (14‒28 days) conditions. (**b**) The MTT assay result of HA, 1%-ZnHA and 2%-ZnHA during culture period. The significant difference was calculated in comparison to the HA group samples

Cell proliferation was determined by measuring the total activity of LDH in the cell lysates and MTT assay. Figure 3A shows that LDH activities of cells throughout the whole culture period, i.e. under both mono-culture and co-culture conditions. As non-proliferating OC had negligible LDH activity, the results mainly represented the proliferation of BMSC/OB in mono-culture and co-culture conditions. Overall, the 1%-ZnHA and 2%-ZnHA samples showed higher LDH activities than those of the HA during the first 13 days. After the addition of RAW264.7 cells on the 13th day, the difference between the Zn doped HA and the pristine HA coatings became small, except that significantly lower LDH value was observed for 2%-ZnHA sample at day 21. We further carried out the MTT assay (Fig. 3B) for cell proliferation analysis. Overall, the 1%-ZnHA and 2%-ZnHA samples had significantly higher OD values than those of the HA samples under mono-culture (2, 4, 7 days) and 1 day after co-culture (14 days), suggesting a stimulating effect of Zn doping on BMSC/OB proliferation. After the Raw264.7 cells were added, there was no significant differences in OD values for the ZnHA and HA samples under co-culture conditions (21, 28 days), despite showing significantly increased OD values.

**Figure 4:**
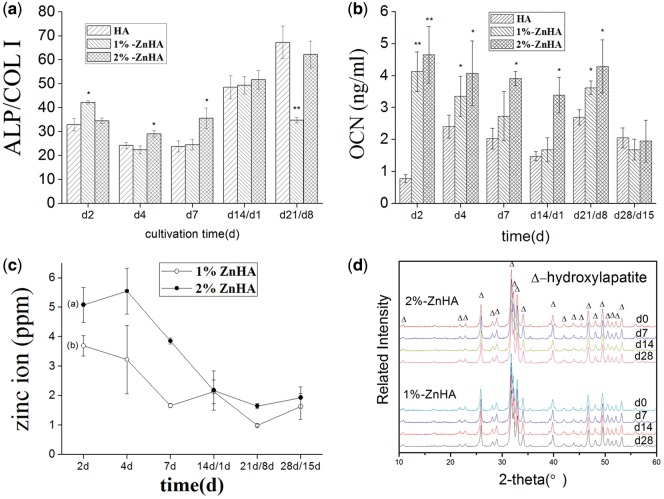
(**a**) The ALP/COL I ratios of BMSC/OB measured under mono-culture (0‒13 days) and co-culture (14‒21 days) conditions. (**a**) OCN secretions of BMSC/OB under mono-culture and co-culture conditions. (**c**) The measured Zn concentrations in cell suspension for 1%-ZnHA and 2%-ZnHA samples. (**d**) X-ray spectra of the ZnHA coatings before (0 day) and after soaking in cell culture medium (7, 14, 28 days). The significant difference was calculated in comparison to the HA group samples

The ALP/COL I ratio can be used as a measurement of differentiation [22]. Since RAW264.7/OC would have little ALP activity, the values were predominantly attributed to the BMSC/OB throughout the culture period. Overall, Fig. 4A shows higher ALP/COL I ratios as a result of the zinc incorporation into the HA lattice, under mono-culture conditions (2, 4 and 7 days), suggesting a stimulating effect of Zn on the osteoblastic differentiation of BMSCs. Similar to the proliferation measurements, the difference became less significant between the HA and the Zn-substituted HA coatings under co-culture conditions, except that significantly lower ALP/COL I ratio was observed for 1%-ZnHA sample at Day 21. The OCN secretion was also analysed throughout the mono-culture and co-culture periods (Fig. 4B). The OCN levels of BMSCs from the two ZnHA (1, 2%) samples were significantly higher than those of the HA samples during all mono-culture times (2, 4, 7 days) and most of the co-culture times (14, 21 days), suggesting a clear stimulating effect of Zn doping on OCN expression.

It has been reported that the zinc content of HA would affect the cellular responses of BMSCs [23]. Figure 4C shows the Zn concentrations in cell suspensions for 1%-ZnHA and 2%-ZnHA samples throughout the culture period. The Zn concentrations were in the ranges of a few ppm. The release of Zn ions from the 1%-ZnHA samples (Fig. 4Cb) was lower than that from the 2%-ZnHA (Fig. 4Ca) at each corresponding culture time. Overall, the dissolution rate of zinc from the ZnHA gradually decreased as a function of time.

### Cell behaviours of OCs in co-culture and mono-culture

**Figure 5:**
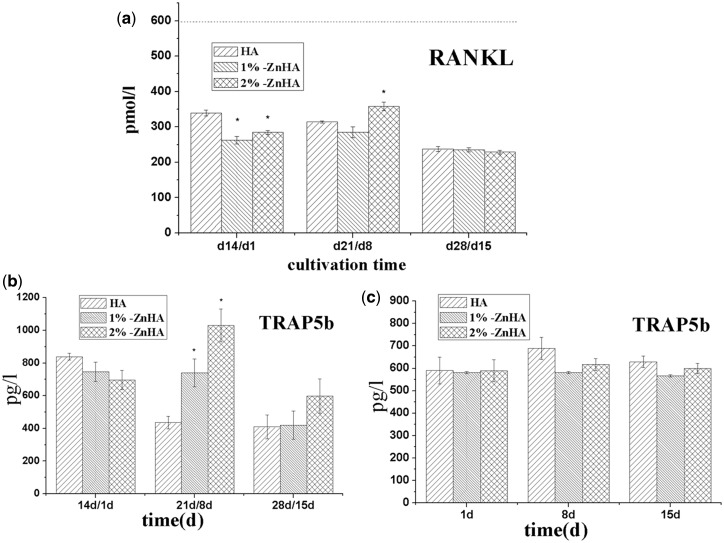
Expressions of RANKL (**a**) and TRAP5b (**b**) under co-culture condition, and TRAP5b (**c**) under mono-culture condition. The significant difference was calculated in comparison to the HA group samples

Two culture conditions were used for analysing the effect of Zn on OC behaviours. Co-culture condition was described previously as in the study of OB behaviours, with RAW264.7 (OC precursors) being added to BMSC derived OB (Fig. 1a). For mono-culture condition, RAW264.7 was cultured with RANKL supplementation (Fig. 1b). The differentiation of the monocytes was evaluated by TRAP activity as well as multi-nuclearity and gene expression of osteoclastic markers. Especially, the TRAP 5b isoform is derived from OCs [24], and the TRAP expression is highly specific to OCs. First, we measured the expression of RANKL in co-culture conditions from d14/d1 to d28/d15 (Fig. 5a). Despite significant decreases of RANKL expressions were observed for the two Zn substituted HA coatings right after the co-culture (d14/d1), no significant differences were observed afterwards at d21/d7 and d28/d15. Figure 5b and c shows the measured TRAP5b activities of the RAW264.7/OC cells in the presence and the absence of the OB, i.e. during the co-culture and mono-culture of RAW264.7/OC, respectively. Under co-culture conditions, TRAP5b expressions of ZnHAs were slight lower than that of the pristine HA right after the initiation of the co-culture (d14/d1). This was not surprising as zinc has been reported to inhibit the OC differentiation. Surprisingly, such trend was reversed and the TRAP5b activities significantly increased in the ZnHA coatings compared with the HA coatings at d21/d8. On the contrary, under mono-culture condition, i.e. with simple RANKL supplements and in the absence of OB, the TRAP5b activities of the ZnHA samples were similar to that of the pristine HA. This suggests that the presence of OB has a profound effect on the OC response to ZnHA, and such effect cannot be reproduced with the simple RANKL supplements.

**Figure 6:**
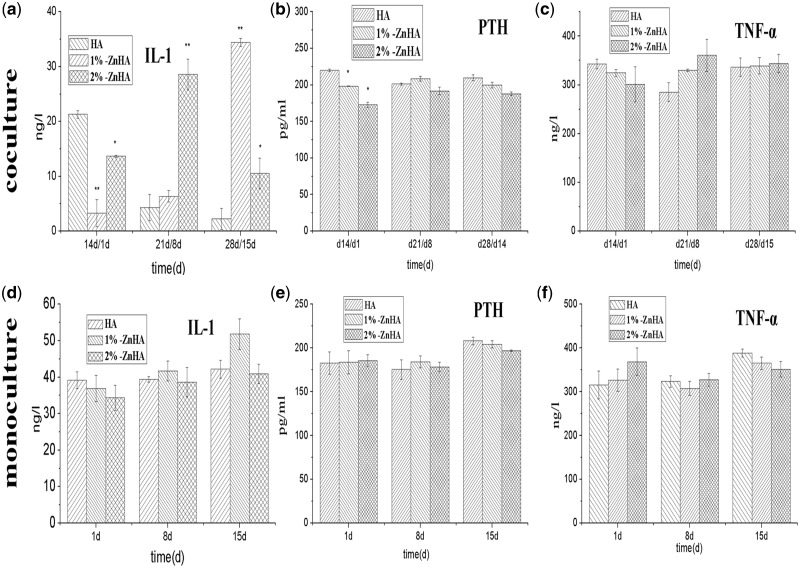
Expressions of IL-1, PTH and TNF-α under co-culture (**a**–**c**) and mono-culture (**d**–**f**) conditions. The significant difference was calculated in comparison to the HA group samples

To further study the surprising modulating effect of ZnHA in the presence of OB, we also monitored the expressions of IL-1, PTH and TNF-α, the other important markers associated with the OC formation and activation, in the presence and the absence of the BMSC/OB. Figure 6a and d shows the IL-1 activity measured during the cultivation time in co-culture and mono-culture RAW264.7/OC. Under co-culture conditions, i.e. in the presence of the BMSC/OB, the IL-1 expressions of the 1%-ZnHA and 2%-ZnHA were significantly lower than that on the HA sample right after co-culture (14 days/1 day). Similar to the TRAP5b activity, such trend was reversed and the IL-1 activities significantly increased in ZnHA coatings compared with the HA coating at d21/d8. As for PTH (Fig. 6b and e) and TNF-α (Fig. 6c and f) activities, while the ZnHA samples had lower expression right after the initiation of the co-culture (14 days/1 day), no reversing was observed afterwards for both activities at d21/d8 and d28/15. Again, in the absence of the BMSC/OB, no significant differences were observed for PTH and TNF-α activities among the three HA samples.

**Figure 7:**
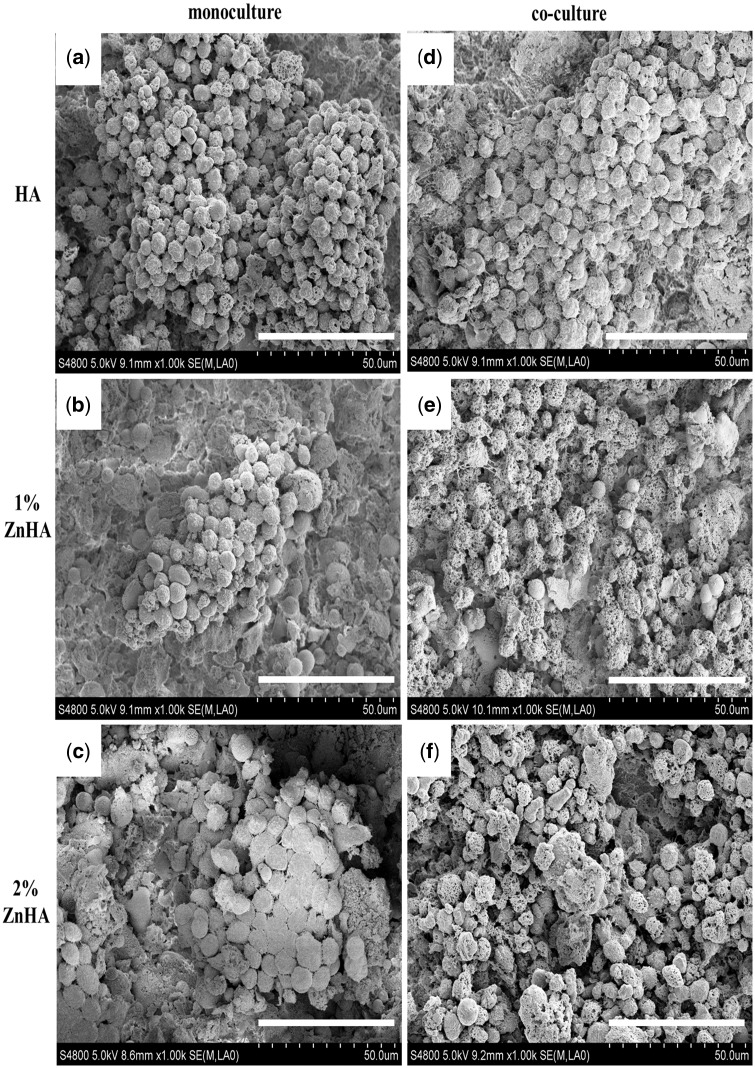
SEM images of cells on d25/d12 of mono-culture (**a**–**c**), and images on d12 of co-culture (**d**–**f**) on HA, 1%- and 2%-ZnHA (bar = 50μm)

The SEM images in Fig. 7 show the morphologies of the cells cultured on different samples at d25/d12. OCs in round form were overwhelmingly observed. The cells on the pristine HA sample displayed an aggregated structure (Fig. 7a). Similar distributions occurred for the two ZnHA samples (Fig. 7b and c). Overall, the RAW264.7/OC showed rounder features and better extension to the material surfaces under the co-culture conditions for the two ZnHAs (Fig. 7e and f) than those under the mono-culture conditions (Fig. 7b and c).

**Figure 8:**
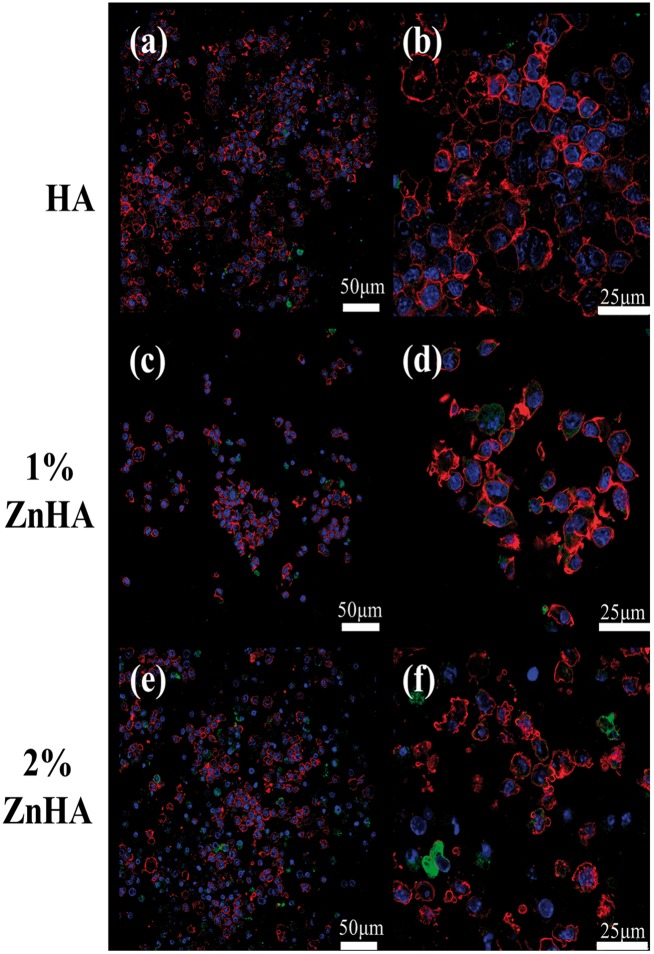
CLSM images of cells cultured for 12 days in mono-culture. The red represents the actin cytoskeleton; green indicates vitronectin receptor α_v_β_3_ integrin and blue represents the nucleolus. Cells on HA (**a**, **b**), 1%-ZnHA (**c**, **d**), 2%-ZnHA (**e**, **f**)

**Figure 9:**
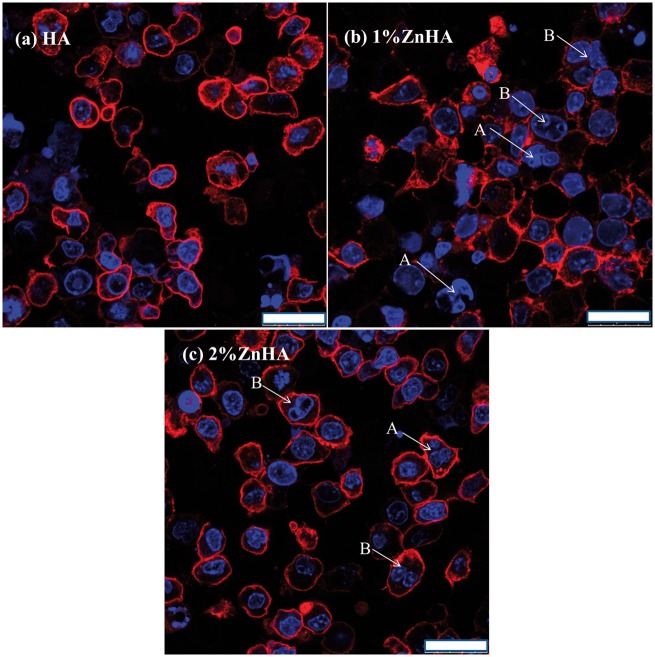
CLSM images of cells after 14 days co-culture. The blue represents the nucleolus. Cells on HA (**a**), 1%-ZnHA (**b**), 2%-ZnHA (**c**), bar = 25μm. The arrows indicate multinucleated cells (A) or nucleus integration (B), respectively

Confocal laser scanning microscope (CLSM) images of the RAW264.7/OC under mono-culture condition (d12) are shown in Fig. 8. There was no clear indication of multi-nuclearity, but actin rings were occasionally observed for all samples. The αvβ3 integrin, a vitronectin receptor protein, was lowly expressed. Overall, no significant differences were found in the phenotypic expressions of the RAW264.7 cultured on three kinds of samples under mono-culture condition. Figure 9 shows the CLSM images of the RAW264.7/OC under co-culture condition (28 days/14 days). There was no clear indication of the presence of the multinucleated cells for the pristine HA samples after 14 days co-culture (Fig. 9a). On the other hand, multinucleated cells or nucleus integration have been occasionally observed for the two ZnHA samples (Fig. 9b and c). The results suggest moderate stimulating effect on OC formation as a result of Zn doping, despite the relatively limited activity for OC formation overall.

## Discussion

Various factors have been reported to affect the bone remodelling process, which involves a critical balance of OB and OC functions. For instance, Zn has a stimulating effect on bone formation through stimulating cell proliferation, ALP activity, collagen synthesis [23] and mineralization *in vitro* and *in vivo*. The effect of Zn on OC differentiation and activity is somehow complicated, but an enhancing effect has been often reported. On the other hand, the OB plays critical role on modulating the OC differentiation through RANKL/RANK/OPG pathway, by means of the binding of RANKL at OB membrane presentation to the membrane receptor RANK on mononuclear OC precursors. The aim of this study was to examine the effect of zinc on OB and OC responses under mono-culture and co-culture conditions (Fig. 1), and in particular the differentiation and activity of OC in the presence and absence of the MSC transformed OBs.

To this end, Zn compositions for incorporation into the HA coatings were selected as 1% and 2%, as both concentrations have been falling in the appropriate composition range that have been reported to have positive influence on cellular responses [25, 26]. The 1% and 2% Zn-HA were successfully prepared through a LPPS process. The XRD spectra suggested that the zinc elements had been successfully incorporated into the HA lattice, as indicated by the respective shifting of the (2 2 1), (1 1 2) and (3 0 0) diffraction peaks. Moreover, the two ZnHA coatings were quite stable during soaking for 28 days, as little change has been found in terms of phase structure (XRD spectra, Fig. 4D) and topographical features (SEM, data not shown here).

The results indicated that Zn exerted positive influence on the proliferation and osteoblastic differentiation of the BMSCs. The LDH activity has been used to partially indicate the cell proliferation [18, 27]. During mono-culture, zinc substitution moderately stimulated the MSC/OB proliferation and differentiation into the osteoblastic lineage, as indicated by the LDH activity and ALP/COL I ratios. This was in agreement with the previous reports [9] despite the enhancement effect observed in our study was slightly weaker than others. Nevertheless, such stimulating effect disappeared under the co-culture condition, i.e. after the addition of RAW264.7 into BMSC/OB. We then used the MTT assay to clarify this issue. The MTT results demonstrated a clear stimulating effect of Zn doping on the BMSC/OB proliferation throughout the mono-culture and early co-culture period. The Zn doping in HA was also found to stimulate the BMSC differentiation, with strongly enhanced OCN secretion and moderately increased ALP/COL I ratio.

Then we monitored the effect of Zn on the OC responses to the HA coatings. A surprising modulation effect of Zn on RAW264.7 cells had been observed under co-culture condition. Right after the addition of the RAW264.7 cells into the BMSC/OB and the switching of the culture medium, ZnHAs showed a moderately inhibitory effect on the TRAP5b activity (Fig. 5b). This was not surprising as Zn has been generally reported to inhibit OC differentiation [13]. However, a surprising reversing effect occurred and the Zn demonstrated a strong stimulating effect on TRAP5b activity afterwards (Fig. 5b). Such strong stimulating effect of Zn on OC differentiation has not been reported previously, to the best of our knowledge. Then we carried out the same experiments in the absence of the BMSC transformed OB, through the addition of RANKL molecule, as the RANKL on the OB membrane is supposed to play the most critical role in modulating OC differentiation through the RANKL/RANK/OPG pathway. However, a different behaviour had been observed for the two ZnHAs, and no stimulating effect of Zn on OC differentiation had been observed. The results strongly suggested that the presence of the OB had a profound effect on modulating the OC response to ZnHA, and both the OB and Zn demonstrated a highly interacted role in modulating the OC functions.

The analyses on IL-1, PTH and TNF-α, other important marker associated with the OC function, might help to shed insight on the interacting role of OB and Zn on OC functions. The IL-1 marker demonstrated a similar pattern as the TRAP5b activity, which was not observed in the PTH and TNF-α analyses. This suggested that IL-1 might have a direct involvement with such surprising modulating effect of Zn on OC function, in the presence of OB instead of a simple RANKL supplementation. IL-1 plays a critical role in OC functions, beside its role in the inflammatory reaction. IL-1 modulates a downstream factor TRAF-6 which in turn modulates both OC formation (though NF-κB and JNK pathways) and activation (through Src pathway) [3]. Zn and BMSC/OB might have a synergistic influence on the up-regulation of the IL-1 expressions. On the other hand, Zn overall had no significant effect on the expressions of TNF-α and PTH under either co-culture or mono-culture condition, despite Zn inhibited the PTH expression on the first day (Fig. 6b) in co-culture. Beside its effect on cell apoptosis, TNF-α is also involved in OC function through a down-streaming factor TRAF-2 which modulates the OC formation through NF-κB and JNK pathways [3]. The PTH, a natural hormone with an important regulation role on OC activity, was apparently not affected by the variation of the culture condition. Nevertheless, the underlying mechanism of the highly interacting role of Zn and OB needs to be further studied.

Such surprising modulation effect of Zn on OC function was also evident on SEM observations. The two ZnHA samples had better OC formation and morphology under co-culture condition (Fig. 7e and f) than under the mono-culture condition (Fig. 7b and c), indicating a stimulating effect of Zn on OC function in the presence of BMSC transformed OB. The CLSM study further confirmed the clear stimulating effect of Zn doping on OC formation only under co-culture condition (Fig. 9b and c), although the nucleus integration and multinucleated cells could only be occasionally seen in the two ZnHA sample and the total activity for OC formation was limited. Perez-Amodio *et al*. [28] found that OBs need to retract firstly and then TRACP^+^ multinucleated cells can be formed within cell-free areas. This might partially contribute to the overall limited activity for OC formation observed for the two ZnHA samples, as the OB lying under the RAW264.7 layer might inhibit the OC formation due to the likely limited OB retraction. Nevertheless, the results suggest that the zinc and the cellular environment likely play highly interacted role in regulating OC differentiation and activity, which might have important implication in understanding the bone remodelling process and improving the treatment of diseases such as osteoporosis.

## Conclusion

In conclusion, we have demonstrated that the cellular environment had a profound effect on the modulating role of Zn in OB and OC differentiation. ZnHA initially showed an inhibitory effect on OC differentiation, and then significantly promoted the OC differentiation at later stages, but only in the presence of BMSC/OB. Such effect was not observed with simple RANKL supplementation in the culture medium, in the absence of co-culture of BMSC/OB with the RAW264.7 cells. The results suggest that the modulating effect of zinc on OC functions is a complicated process and the presence of the OB likely plays a highly interacted role with zinc in modulating the OB and OC activities, thus affecting the bone remodelling process.

## Funding

The present research was supported by the Natural Science Foundation of China (No. 31170922), National Basic Research Program of China (No. 2012cb619103), the Research Fund for the Doctoral Program of Higher Education (No. 20120181110058), and Program for New Century Excellent Talents in University (NCET-12-0387).

*Conflict of interest statement.* None declared.

## References

[1] Harada S, Rodan GA. Control of osteoblast function and regulation of bone mass. Nature 2003;423:349–55. 12748654 10.1038/nature01660

[2] Chien KR, Karsenty G. Longevity and lineages: toward the integrative biology of degenerative diseases in heart, muscle, and bone. Cell 2005;120:533–44. 15734685 10.1016/j.cell.2005.02.006

[3] Wright HL, McCarthy HS, Middleton J et al RANK, RANKL and osteoprotegerin in bone biology and disease. Curr Rev Musculoskelet Med 2009;2:56–64. 19468919 10.1007/s12178-009-9046-7 PMC2684955

[4] Tapiero H, Townsend DM, Tew KD. Trace elements in human physiology and pathology. Copper. Biomed Pharmacother 2003;57:386–98. 14652164 10.1016/s0753-3322(03)00012-x PMC6361146

[5] Hsieh HS, Navia JM. Zinc deficiency and bone formation in guinea pig alveolar implants. J Nutr 1980;110:1581–8. 7400848 10.1093/jn/110.8.1581

[6] Oner G, Bhaumick B, Bala RM. Effect of zinc deficiency on serum somatomedin levels and skeletal growth in young rats. Endocrinology 1984;114:1860–3. 6714170 10.1210/endo-114-5-1860

[7] Yamaguchi M, Yamaguchi R. Action of zinc on bone metabolism in rats. Increases in alkaline phosphatase activity and DNA content. Biochem Pharmacol 1986;35:773–7. 3954786 10.1016/0006-2952(86)90245-5

[8] Hall SL, Dimai HP, Farley JR. Effects of zinc on human skeletal alkaline phosphatase activity in vitro. Calcif Tissue Int 1999;64:163–72. 9914326 10.1007/s002239900597

[9] Hashizume M, Yamaguchi M. Stimulatory effect of beta-alanyl-l-histidinato zinc on cell proliferation is dependent on protein synthesis in osteoblastic MC3T3-E1 cells. Mol Cell Biochem 1993;122:59–64. 8350864 10.1007/BF00925737

[10] Hashizume M, Yamaguchi M. Effect of beta-alanyl-l-histidinato zinc on differentiation of osteoblastic MC3T3-E1 cells: increases in alkaline phosphatase activity and protein concentration. Mol Cell Biochem 1994;131:19–24. 8047061 10.1007/BF01075720

[11] Yamaguchi M, Weitzmann MN. Zinc stimulates osteoblastogenesis and suppresses osteoclastogenesis by antagonizing NF-κB activation. Mol Cell Biochem 2011;355:179–86. 21533765 10.1007/s11010-011-0852-z

[12] Yamada Y, Ito A, Kojima H et al Inhibitory effect of Zn^2+^ in zinc-containing beta-tricalcium phosphate on resorbing activity of mature osteoclasts. J. Biomed Mater Res A 2008;84:344–52. 17618520 10.1002/jbm.a.31265

[13] Roy M, Fielding GA, Bandyopadhyay A et al Effects of zinc and strontium substitution in tricalcium phosphate on osteoclast differentiation and resorption. Biomater Sci 2013;1:74–82. 10.1039/C2BM00012A PMC3825406 24244866

[14] Khadeer MA, Sahu SN, Bai G et al Expression of the zinc transporter ZIP1 in osteoclasts. Bone 2005;37:296–304. 16005272 10.1016/j.bone.2005.04.035

[15] Calhoun NR, Smith JC Jr, Becker KL. The role of zinc in bone metabolism. Clin Orthop Relat Res 1974;103:212–34. 10.1097/00003086-197409000-00084 4213480

[16] Holloway WR, Collier FM, Herbst RE et al Osteoblast-mediated effects of zinc on isolated rat osteoclasts: inhibition of bone resorption and enhancement of osteoclast number. Bone 1996;19:137–42. 8853857 10.1016/8756-3282(96)00141-x

[17] Li X, Senda K, Ito A et al Effect of Zn and Mg in tricalcium phosphate and in culture medium on apoptosis and actin ring formation of mature osteoclasts. Biomed Mater 2008;3:045002. 18824780 10.1088/1748-6041/3/4/045002

[18] Heinemann C, Heinemann S, Worch H et al Development of an osteoblast/osteoclast co-culture derived by human bone marrow stromal cells and human monocytes for biomaterials testing. Eur Cell Mater 2011;21:80–93. 21267944 10.22203/ecm.v021a07

[19] Birmingham E, Niebur GL, McHugh PE et al Osteogenic differentiation of mesenchymal stem cells is regulated by osteocyte and osteoblast cells in a simplified bone niche. Eur Cell Mater 2012;23:13–27. 22241610 10.22203/ecm.v023a02

[20] Huang T, Xiao YF, Wang SL et al Nanostructured Si, Mg, ${\text{CO}}_{3}^{2‒}$ substituted hydroxyapatite coatings deposited by liquid precursor plasma spraying: synthesis and characterization. J. Therm Spray Technol 2011;20:829–36.

[21] Huang Y, Song L, Huang T et al Characterization and formation mechanism of nano-structured hydroxyapatite coatings deposited by the liquid precursor plasma spraying process. Biomed Mater 2010;5:054113. 10.1088/1748-6041/5/5/054113 20876965

[22] Botelho CM, Brooks RA, Best SM et al Human osteoblast response to silicon-substituted hydroxyapatite. J. Biomed Mater Res A 2006;79:723–30. 16871624 10.1002/jbm.a.30806

[23] Ito A, Otsuka M, Kawamura H et al Zinc-containing tricalcium phosphate and related materials for promoting bone formation. Curr Appl Phys 2005;5:402–6.

[24] Halleen JM, Ylipahkala H, Alatalo SL et al Serum tartrate-resistant acid phosphatase 5b, but not 5a, correlates with other markers of bone turnover and bone mineral density. Calcif Tissue Int 2002;71:20–5. 12073156 10.1007/s00223-001-2122-7

[25] Grandjean-Laquerriere A, Laquerriere P, Jallot E et al Influence of the zinc concentration of sol-gel derived zinc substituted hydroxyapatite on cytokine production by human monocytes in vitro. Biomaterials 2006;27:3195–200. 16487585 10.1016/j.biomaterials.2006.01.024

[26] Chen Y, Wang J, Zhu XD et al The directional migration and differentiation of mesenchymal stem cells toward vascular endothelial cells stimulated by biphasic calcium phosphate ceramic. Regen Biomater 2018;5:129–39. 29977596 10.1093/rb/rbx028 PMC6007427

[27] Haslam G, Wyatt D, Kitos PA. Estimating the number of viable animal cells in multi-well cultures based on their lactate dehydrogenase activities. Cytotechnology 2000;32:63–75. 19002967 10.1023/A:1008121125755 PMC3449446

[28] Perez-Amodio S, Beertsen W, Everts V. ( Pre-) osteoclasts induce retraction of osteoblasts before their fusion to osteoclasts. J. Bone Miner Res 2004;19:1722–31. 15355568 10.1359/JBMR.040509

